# Design and Characterization of Recombinant and Chimeric BoNT/A Neurotoxins with Receptor-Binding Domain Grafting

**DOI:** 10.3390/toxins18050205

**Published:** 2026-04-29

**Authors:** Sihan Pan, Yuanzhi Ye, Yang Li, Hongxin Fu, Jufang Wang

**Affiliations:** 1 School of Biology and Biological Engineering, South China University of Technology, Guangzhou 510006, China; 2 Guangdong Provincial Key Laboratory of Fermentation and Enzyme Engineering, South China University of Technology, Guangzhou 510006, China

**Keywords:** botulinum toxin type A, drug design, chimeric protein, neurotoxin potency, duration

## Abstract

Botulinum neurotoxins (BoNTs) act on peripheral cholinergic nerve terminals, inducing reversible muscle paralysis and profound therapeutic effects. However, their limited cell-type specificity and narrow therapeutic window have motivated the development of engineered variants. Here, a modular strategy was employed to construct full-length chimeric BoNTs, grafting receptor-binding segments from BoNT/B or BoNT/F onto the BoNT/A framework. The novel chimeras AAAF and AAFF efficiently cleaved rSNAP-25 in cell-free assays. Firstly, both toxins showed effective cellular uptake and cleaved endogenous SNAP-25 in Neuro-2a cells, with cleavage efficiencies of approximately 46% for AAAF and 73% for AAFF, highlighting the enhanced activity of AAFF. Secondly, AAAF induced faster recovery from reversible muscle paralysis compared to rBoNT/A-WT, whereas AAFF produced more sustained paralysis, with both exhibiting reduced systemic toxicity. Despite these altered pharmacological profiles, the chimeras required higher doses than rBoNT/A-WT to induce neuromuscular effects. Collectively, this study presents the design of novel chimeric BoNT/A-F proteins, characterizes their functional activities, and provides a preliminary exploration of how domain grafting affects cellular uptake, enzymatic activity, and neuromuscular pharmacodynamics.

## 1. Introduction

Botulinum neurotoxins (BoNTs) are potent protein toxins produced primarily by the strictly anaerobic *Clostridium botulinum*, as well as by related *Clostridium* species such as *C. butyricum* and *C. baratii* [1]. They induce flaccid paralysis by selectively binding peripheral cholinergic nerve terminals and blocking acetylcholine release [2,3]. Owing to their exceptionally high specificity, BoNTs exhibit profound therapeutic effects at minute, locally administered doses and are now standard-of-care agents for dystonia, chronic migraine, overactive bladder, and an expanding list of other disorders [4,5,6,7,8]. Traditionally, BoNTs are classified into seven antigenically distinct serotypes (A-G), and several novel botulinum neurotoxins, including BoNT/X and BoNT/H, have also been identified [9,10]. Among these, serotype A (BoNT/A) has garnered the greatest clinical and research interest. Large-scale genome sequencing has revealed more than 45 functional subtypes, many of which display marked amino acid sequence divergence and may exhibit distinct pharmacological properties [11]. To date, a growing number of naturally occurring chimeric toxins have been identified. A prototypical example is BoNT/CD, whose light chain and translocation domain (LC-H_N_) originate from BoNT/C and whose receptor-binding domain (H_C_) is derived from BoNT/D, with its recombinant form showing enhanced neuronal activity, strong local paralysis, and lower systemic toxicity, highlighting the role of domain composition [12]. Together with emerging subtypes and BoNT-like proteins, these chimeras enlarge the BoNT superfamily and constitute an invaluable natural and synthetic library for next-generation therapeutic development [13]. Detailed structural analyses of their sequence and conformational diversity now provide the molecular blueprint required to engineer variants with enhanced potency, refined specificity, and broader clinical indications.

Each BoNT isoform is produced as a single-chain precursor that folds into a 150 kDa polypeptide composed of a 50 kDa light chain (LC) and a 100 kDa heavy chain (HC). A conserved inter-chain disulfide bond, together with a belt loop protruding from the heavy chain and maintained by the light chain, locks this configuration until bacterial or host proteases excise an internal nick, releasing the fully active di-chain toxin [14]. BoNTs are architecturally organized into three functional domains: (i) the catalytic light chain (LC, 50 kDa), a zinc-dependent metalloprotease that cleaves SNARE (soluble N-ethylmaleimide-sensitive factor attachment protein receptor) proteins essential for synaptic vesicle fusion; (ii) the N-terminal translocation domain (H_N_, 50 kDa), which orchestrates pH-triggered translocation of the LC into the cytosol; and (iii) the receptor-binding domain (H_C_, 50 kDa), comprising H_CN_ and H_CC_ subdomains that confer highly specific recognition of neuronal surface receptors [15,16,17]. Neurotoxicity at motor-nerve terminals and neuromuscular junctions is initiated by a sequential, dual-receptor engagement [18]. The H_CC_ subdomain initially binds polysialylated gangliosides (PSGs), which serve as molecular “antennae” that enrich the toxin at the presynaptic surface [19,20]. This low-affinity contact is followed by a high-affinity interaction between the H_C_ N-terminal segment (H_CN_) and the luminal domain of synaptic vesicle glycoproteins SV2 (SV2A, SV2B, or SV2C), which mediates efficient endocytosis into both hyperactive and less active nerve terminals [21,22]. Overall, natural botulinum neurotoxins are potent yet limited by narrow target specificity and a restricted therapeutic index, with generally low immunogenicity. Among them, BoNT/A selectively cleaves SNAP-25, efficiently engages its receptors, and is widely used clinically, whereas BoNT/B shows relatively higher immunogenic potential [23].

To overcome these challenges, engineered BoNTs with enhanced stability, reduced immunogenicity, and tunable functionality are now being systematically developed to expand their clinical repertoire. Chimeric botulinum toxins, constructed by recombining functional domains from different serotypes, represent a promising platform for expanding therapeutic applications, yet their rational optimization remains in its infancy. Previous studies have revealed two complementary engineering paradigms: (i) Translocation-domain chimerism enhances pharmacodynamic properties by modulating the pH-dependent membrane translocation process. Replacing the BoNT/A light-chain-translocation module (LC-H_N_) with its BoNT/E counterpart (chimera EA) accelerated cytosolic delivery by leveraging the higher pH sensitivity of the BoNT/E moiety, whereas chimera AE prolongs neuromuscular paralysis. These data underscore the pivotal role of translocation-domain pH responsiveness in modulating onset and duration [24,25]. (ii) Receptor-binding domain substitution boosts potency by enhancing binding affinity to synaptic vesicle receptors and promoting efficient cellular uptake. Chimeras bearing the BoNT/B receptor-binding domain (AAAB and AABB) exhibit ~4-fold higher activity in mouse phrenic nerve assays [26]. Given that hemidiaphragms from Balb/c mice are ~7-fold more sensitive to BoNT/A than BoNT/B, the enhanced ex vivo activity of BoNT/A-B chimeras likely reflects receptor-binding domain recombination, with its B-type domain engaging synaptotagmin I/II to promote cellular uptake [27]. However, in the Digit Abduction Score (DAS) assay, Wang et al. reported that their BoNT/AB chimera, despite reduced specific toxicity, produced an approximately 1.7-fold longer period of neuromuscular paralysis than wild-type BoNT/A [28]. Subsequent studies also observed increased ex vivo potency of AABB, whereas independent in vivo investigations reported potency and duration comparable to wild-type BoNT/A [29]. These discrepancies may arise from differences in protein dose, limitations of the DAS assay, and other experimental factors. Overall, AABB may retain the potency and duration of BoNT/A while incorporating the autonomic targeting properties of BoNT/B, suggesting potential advantages for acetylcholine-mediated autonomic disorders.

The chimeric toxins exhibit enhanced pharmacological properties through domain recombination, demonstrating promising therapeutic potential that warrants further systematic investigation. However, current research has been limited to serotypes A, B, and E, whereas BoNT/A—which is clinically dominant—binds synaptosomes more weakly than other serotypes, underscoring untapped opportunities for further optimization. Unlike its relatives, BoNT/F recognizes multiple SV2 isoforms and retains the ganglioside-binding motifs of BoNT/A, yet BoNT/A-F chimeras remain largely unexplored [30]. To address these issues, we therefore aim to rationally design and characterize a panel of BoNT/A-F chimeras with the goals of strengthening receptor interactions, boosting functional efficacy, and expanding the therapeutic application of botulinum toxins. Here, we engineered chimeric BoNTs by grafting the receptor-binding segments derived from BoNT/B or BoNT/F onto the BoNT/A scaffold. The resultant constructs allowed us to dissect, in a stepwise manner, how swapping exogenous binding domains affects toxin expression, structural stability, catalytic efficiency, receptor-mediated uptake, and neuromuscular blockade. Comprehensive in vitro biochemical, cellular uptake, substrate-cleavage, and in vivo functional analyses collectively characterized the activity profiles of the chimeras.

## 2. Results

### 2.1. Design, Recombinant Expression, and Purification of Toxins

Although BoNT/A exhibits the highest catalytic potency among serotypes, its modest affinity for synaptosomes limits neuronal uptake and, consequently, the therapeutic index [26]. To reconcile catalytic power with cellular entry, chimeric toxins have been engineered by grafting the receptor-binding segments of serotypes B or E onto the BoNT/A scaffold, yielding a series of chimeras with enhanced neuronal targeting, improved intracellular delivery, and—in some cases—prolonged neuromuscular blockade [24,25,28,29,31]. This domain-swapping strategy is enabled by the high structural homology shared across serotypes, allowing modular exchange without compromising overall structural integrity. BoNT/F, which recognizes all SV2 isoforms and retains a ganglioside-binding motif conserved with the BoNT/A, is an attractive yet under-utilized donor for such chimeras [30]. Here, we design and characterize a series of BoNT/A-F chimeras that aim to overcome the uptake-efficiency bottleneck of wild-type BoNT/A and broaden therapeutic options.

**Figure 1:**
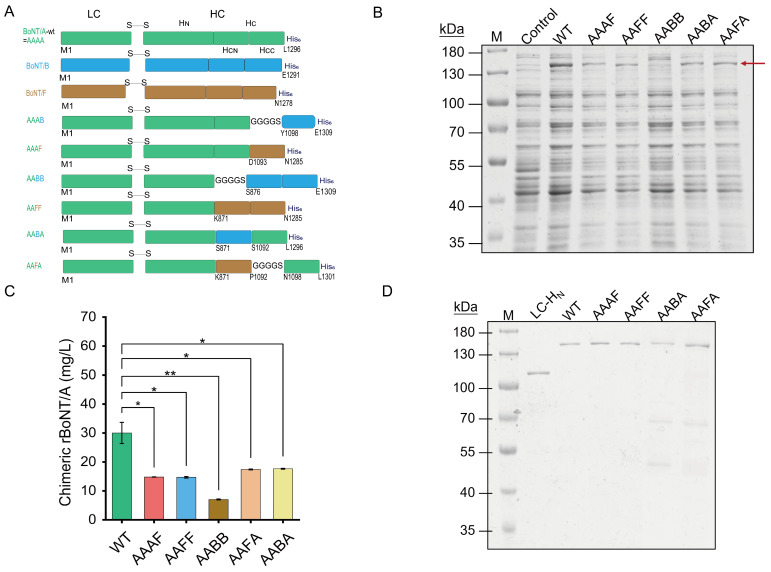
Design and preparation of chimeric BoNTs: (**A**) Schematic diagram of chimeric rBoNT/A constructs. Domain replacements were performed using sequences from BoNT/B and BoNT/F to generate six chimeric variants: AAAB, AAAF, AAFF, AABB, AABA, and AAFA. (**B**) SDS-PAGE analysis. M: marker; control: uninduced sample. The arrow indicates the full-length toxins. (**C**) Protein yield. The production level of each chimeric toxin was measured, providing a direct comparison to rBoNT/A-WT. (**D**) The purification of BoNT/A chimeric toxins by Ni-NTA affinity chromatography. Each experiment was performed in three independent repeats. Data are presented as mean ± standard deviation (SD), with statistical analysis by one-way ANOVA (0.01 < * *p* ≤ 0.05, 0.001 < ** *p* ≤ 0.01).

Under this premise, a panel of full-length chimeric proteins was designed by systematically swapping the receptor-binding domains portions (H_CN_, H_CC_, or H_C_) of BoNT/B or BoNT/F into the BoNT/A scaffold while preserving its catalytic domain (LC) and the translocation domain (H_N_) (Figure 1A). Six constructs were produced: AAAB and AAAF (H_CC_ replaced); AABA and AAFA (H_CN_ replaced); and AABB and AAFF (entire H_C_ replaced). In this nomenclature, each capital letter corresponds to the serotype origin of the LC, H_N_, H_CN_, and H_CC_ domains, respectively. To maximize expression and solubility, a flexible linker (GGGGS) was introduced at domain junctions, a strategy repeatedly shown to preserve the structure and function of multi-domain proteins [32]. This modular approach offers a versatile platform for dissecting the contributions of individual receptor-binding domains and for tuning neuronal specificity.

The expression of recombinant wild-type BoNT/A (rBoNT/A-WT) was systematically optimized for the temperature, IPTG concentration, and medium composition, and the titer reached up to 30.02 mg/L, achieved under 0.5 mM IPTG in TB medium at 16 °C for 24 h; large-scale fermentation produced ~9 mg/L purified toxin, with an overall recovery of approximately 30.3% (Figure S1A–E). The lower purified yield reflects typical losses during the purification and scaling-up, yet is comparable to the 8 mg/L previously reported for enzymatically deactivated recombinant BoNT/A expressed in *E. coli* BL21 (DE3) under non-optimized conditions, underscoring that systematic optimization and efficient purification enable robust, scalable production of recombinant BoNT variants [33].

Then, the optimized protocols were applied to the production of BoNT/A-based chimeric toxins. Among them, the majority of AAAB accumulated in insoluble bodies, and even the insertion of a GGGGS linker failed to rescue solubility, indicating pronounced structural incompatibility (Figure S2A). In contrast, the remaining five chimeric toxins (AAAF, AAFF, AABB, AABA, and AAFA) were recovered in the soluble fraction at variable yields (Figure 1B). Densitometric quantification, normalized to rBoNT/A-WT, revealed the relative expression levels of approximately 50% for AAAF and AAFF, 60% for AAFA and AABA, and 23% for AABB (Figure 1C). Notably, the BoNT/A-F chimeras achieved soluble expression even without a flexible linker, suggesting better inter-domain compatibility than the BoNT/A-B combination. The AABB protein, however, showed a low soluble yield and poor purification efficiency, and high-purity preparations could only be obtained after size-exclusion chromatography; therefore, this construct was not included in the subsequent functional analyses.

Similarly, these four chimeric toxins (AAAF, AAFF, AABA, and AAFA) were upscaled and subjected to nickel affinity chromatography. Compared with the wild-type, all chimeras exhibited lower expression levels and reduced recovery after purification; for example, AAAF eluted at a higher imidazole concentration but yielded less overall protein (Figure S2B,C). The BCA assay and ImageJ (version 1.53k) densitometry of the final eluates indicated protein concentrations in the range of 0.01–0.05 mg/mL for all chimeric constructs, with batch-to-batch variability across independent preparations, while maintaining sufficient purity for the functional assays (Figure 1D; Table S1). Concentration differences were normalized prior to functional testing. Collectively, these data underscore that domain compatibility dictates both expression efficiency and purification performance, thereby confirming that functional chimeric toxins can nevertheless be produced at a preparative scale.

### 2.2. Trypsin-Mediated Activation and Proteolytic Activity of Chimeric BoNTs in Cell-Free Systems

**Figure 2:**
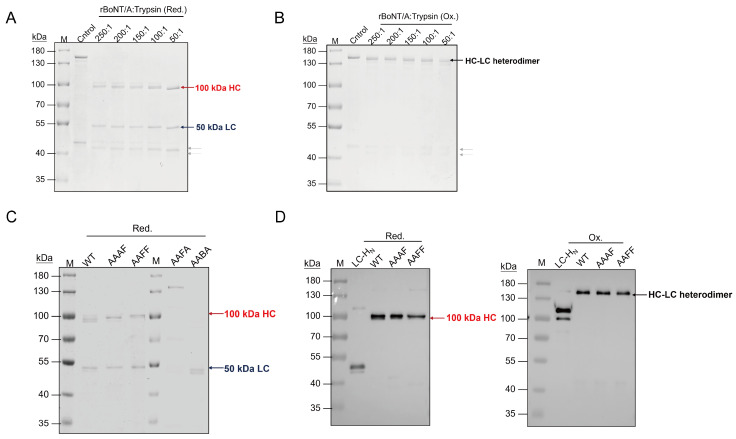
Trypsin-mediated activation evaluation of chimeric BoNTs: (**A**,**B**) SDS-PAGE analysis of the rBoNT/A-WT treated with varying trypsin concentrations. The arrows labeled by red, blue, black, and gray indicate 100 kDa HC, 50 kDa LC, and 150 kDa HC-LC heterodimer, and undesired species or host-cell-derived contaminants, respectively. M: molecular weight marker; control: the concentrated protein after ultrafiltration. (**A**) Reducing conditions. The samples were denatured with β-mercaptoethanol and heated at 95 °C for 10 min. (**B**) Non-reducing conditions. (**C**) SDS-PAGE analysis of rBoNT/A-WT and the chimeric toxins after trypsin activation (toxin: trypsin = 100:1, 37 °C for 60 min) under reducing conditions. (**D**) Western blot analysis of rBoNT/A-WT and the chimeric toxins after activation under reducing and non-reducing conditions.

The BoNTs are expressed as inactive 150 kDa single-chain precursors that are proteolytically cleaved between the light and heavy chains to yield active 100 kDa HC and 50 kDa LC heterodimers. Under reducing conditions, trypsin-digested rBoNT/A-WT generated the expected, separated HC and LC bands (Figure 2A). In contrast, non-reducing SDS-PAGE analysis revealed the cleaved product remained as an intact, disulfide-linked HC-LC heterodimer (~150 kDa) (Figure 2B). A titration of trypsin showed that higher enzyme: substrate ratios sharpened the HC/LC bands and drove the reaction to completion; however, excessive trypsin promoted over-digestion. Consequently, a ratio of 100:1 (rBoNT/A to trypsin, *w*/*w*) was selected as the optimal and adopted for all subsequent activations. Since the chimeras retain the native LC-HC linker, these conditions are expected to activate them with equal efficiency.

Subsequently, the chimeras (AAAF, AAFF, AABA, and AAFA) were subjected to the same activation procedure (Figure 2C). Under reducing conditions, AAAF and AAFF exhibited two distinct bands (~100 kDa HC and ~50 kDa LC), consistent with the wild-type pattern, indicating that trypsin efficiently cleaved the inter-chain linker without disrupting the disulfide bond, while the faint ~50 kDa bands likely represent minor species or partially processed forms. In contrast, AABA only produced a single 50 kDa band, indicating extensive HC degradation, while AAFA remained almost entirely intact, suggesting strong resistance to trypsin. These results demonstrate that the domain substitutions profoundly altered both structural stability and protease susceptibility, and may also reflect limited accessibility of trypsin to the native cleavage sites. To further verify the cleavage pattern, Western blot analysis using an anti-His antibody was performed on rBoNT/A-WT and the chimeras AAAF and AAFF. Under reducing conditions, the His signal was mainly detected on the heavy chain, whereas under non-reducing conditions, it appeared predominantly as a full-length band, indicating a disulfide-linked heterodimeric structure. Given that the functional activity requires the LC-HC heterodimer, AAAF and AAFF were selected for the subsequent bioassays.

**Figure 3:**
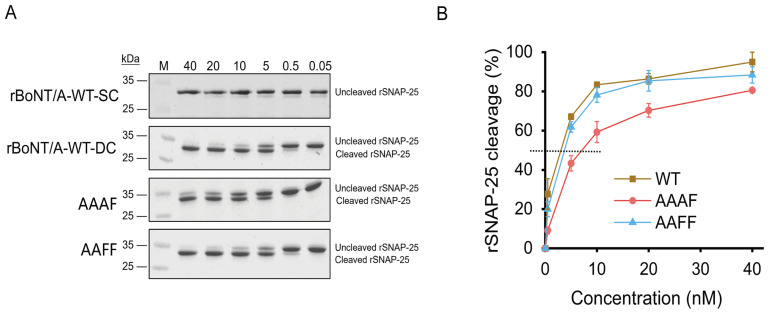
Cell-free proteolytic activity assay: (**A**) SDS-PAGE analysis of recombinant SNAP-25 (rSNAP-25) cleavage by di-chain rBoNT/A-WT (rBoNT/A-WT-DC), single-chain rBoNT/A-WT (rBoNT/A-WT-SC), AAAF, and AAFF. Reactions were performed at 37 °C with the indicated toxin concentrations (0.05, 0.5, 5, 10, 20, and 40 nM) and 2.4 μM rSNAP-25 in assay buffer for 30 min. Cleavage efficiency was determined by densitometric quantification of the cleaved SNAP-25 band, normalized to the total SNAP-25 signal (intact + cleaved) in each lane. (**B**) Concentration–response curves of SNAP-25 cleavage derived from the densitometric data in panel A, showing concentration-dependent substrate cleavage for each toxin variant. Dashed line: ~50% rSNAP-25 cleavage.

To assess the catalytic activity of rBoNT/A-WT and chimeric toxins, a cell-free proteolytic assay was conducted using recombinant rSNAP-25 as the substrate. BoNT/A specifically cleaves SNAP-25 at the Q197-R198 bond, disrupting the SNARE complex and thereby blocking acetylcholine release. The 6 × His-tagged rSNAP-25 substrate, expressed in *E. coli* and purified via the nickel affinity chromatography (Figure S3), was incubated with either activated rBoNT/A-WT (rBoNT/A-WT-DC) or single-chain form (rBoNT/A-WT-SC) toxins. Only the activated heterodimer generated the desirable cleavage fragment, whereas the single-chain precursor was inactive (Figure 3A), confirming both toxin functionality and assay specificity.

All subsequent assays were conducted using the activated di-chain (DC) forms of rBoNT/A-WT and the chimeric toxins AAAF and AAFF to ensure accurate assessment of catalytic activity. The SDS-PAGE assay resolved the intact 25 kDa rSNAP-25 from its slightly smaller cleavage product, enabling unambiguous quantification of proteolysis. A concentration-dependent SNAP-25 cleavage assay was performed using toxin concentrations ranging from 0.5 to 40 nM, a range within which effective substrate cleavage was observed (Figure 3A,B). Notably, AAFF exhibited a cleavage profile comparable to rBoNT/A-WT, indicating that the chimeric toxin retained catalytic activity similar to the wild-type, whereas AAAF showed slightly reduced efficiency, with trace SNAP-25 processing still detectable at relatively higher concentrations. These results demonstrate that both chimeric toxins maintain in vitro proteolytic activity toward SNAP-25, with AAFF comparable to the wild-type and AAAF displaying a modest reduction. As the catalytic (LC) and translocation (H_N_) domains were identical to those of BoNT/A, these observed differences likely arise from subtle conformational changes introduced by replacement of the receptor-binding domain, which may modulate substrate accessibility or enzymatic stability. Overall, domain replacement alters enzymatic activity without compromising catalytic function, supporting further mechanistic and functional studies of the chimeric toxins.

### 2.3. Intracellular Uptake and Proteolytic Activity of Chimeric BoNTs in Neuro-2a Cells

To comprehensively evaluate the cellular behavior and potency of the chimeric toxins, the Neuro-2a cells were employed as an in vitro model owing to their neuronal characteristics and well-documented sensitivity to botulinum neurotoxins [34]. However, it should be noted that Neuro-2a cells, although exhibiting neuron-like morphology and basic synaptic properties, express only limited amounts of complex gangliosides (e.g., GM1 and GT1b) and lack the subtype-specific molecular features characteristic of primary neurons [34,35]. These limitations may lead to an underestimation of the cellular uptake and functional potency of certain chimeric toxins, particularly those that depend on specific ganglioside or receptor interactions for efficient internalization.

**Figure 4:**
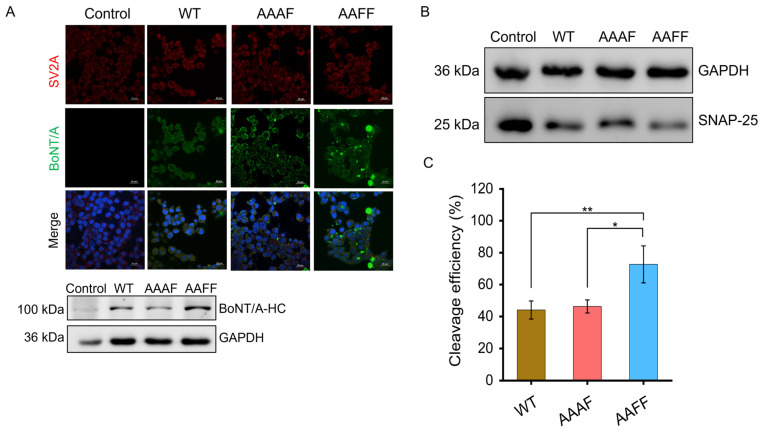
Evaluation of cellular uptake and intracellular enzymatic activity: (**A**) Immunofluorescence and Western blot analysis of toxin internalization in Neuro-2a cells treated with His-tagged rBoNT/A-WT, AAAF, and AAFF (10 nM) for 12 h. Upper panels show representative confocal images illustrating intracellular green fluorescence corresponding to the His-tagged toxin heavy chain detected with an anti-His primary antibody and an Alexa Fluor 488-conjugated secondary antibody. Scale bar = 20 μm. Lower panels show Western blot analysis of cell lysates using an anti-His antibody to detect the internalized toxin heavy chain, providing qualitative confirmation of cellular uptake. (**B**) Western blot analysis of endogenous SNAP-25 cleavage in Neuro-2a cells after 12 h incubation with 10 nM activated di-chain rBoNT/A-WT, AAAF, or AAFF. (**C**) Densitometric quantification of SNAP-25 cleavage following 12 h treatment. Cleavage efficiency was determined by ImageJ-based densitometric analysis, with band intensities normalized to GAPDH as a loading control. Data represent three independent experiments and were analyzed by one-way ANOVA. Results are shown as mean ± standard deviation (SD). Statistical significance is indicated as: 0.01 < * *p* ≤ 0.05, 0.001 < ** *p* ≤ 0.01.

Immunofluorescence staining assays were performed to visualize toxin internalization, utilizing His-tag antibodies for the toxins and DAPI for nuclear counter-staining. Neuro-2a cells were incubated for 12 h with 10 nM activated di-chain forms of rBoNT/A-WT or the chimeric toxins AAAF and AAFF. First, fluorescence microscopy revealed clear intracellular green signals in cells treated with both the wild-type and chimeric toxins, indicating efficient internalization of the His-tagged toxin heavy chain. Second, Western blot analysis of cellular lysates further confirmed toxin uptake, as the His-tagged heavy chain was detected in the intracellular protein fraction following treatment (Figure 4A). In addition, intracellular enzymatic activity was evaluated by analyzing endogenous SNAP-25 cleavage under the same conditions (Figure 4B). All toxins induced detectable SNAP-25 processing, with cleavage efficiencies of approximately 44% for rBoNT/A-WT, 46% for AAAF, and 73% for AAFF. Collectively, these results indicate that both chimeric toxins are efficiently internalized and retain intracellular proteolytic activity, with the AAFF exhibiting enhanced SNAP-25 processing under the conditions tested.

Since the light chain of chimeric toxins is identical to the rBoNT/A-WT, their intracellular activity stems primarily from domain replacement and the grafting of large functional regions. Immunofluorescence and Western blot analyses confirmed efficient uptake of both AAAF and AAFF into Neuro-2a cells and the preservation of intracellular proteolytic activity. Together, these results demonstrate that the chimeric toxins remain functionally active while highlighting the limitations of the Neuro-2a model for assessing engineered botulinum neurotoxins. Importantly, the cellular data provide a foundation for further evaluation of their in vivo functional activity.

### 2.4. Assessment of Digit Abduction Score (DAS) in Mice

To evaluate the in vivo neuromuscular potency of the chimeric toxins, the Digit Abduction Score (DAS) assay was performed in a murine model—a clinically relevant, ethically refined alternative to the traditional LD_50_ test that quantifies toxin-mediated inhibition of reflexive digit abduction after intramuscular injection [36,37]. Dosing for the rBoNT/A-WT (5 pg/mouse) was adopted from previous studies [38,39]. To initially compare with the wild-type toxin, AAAF and AAFF were first administered at the same dose; however, this dosage failed to induce significant muscle paralysis. Therefore, escalating-dose experiments were subsequently conducted to determine the minimal paralytic thresholds of the chimeric toxins. As summarized in Table S4, AAAF at 10 ng/mouse and AAFF at 1 ng/mouse evoked pronounced muscle paralysis (DAS: 3–4) of the right gastrocnemius muscle within 8 h, confirming potent local neuromuscular blockade. The empirically determined doses were then used in all subsequent in vivo assessments.

**Figure 5:**
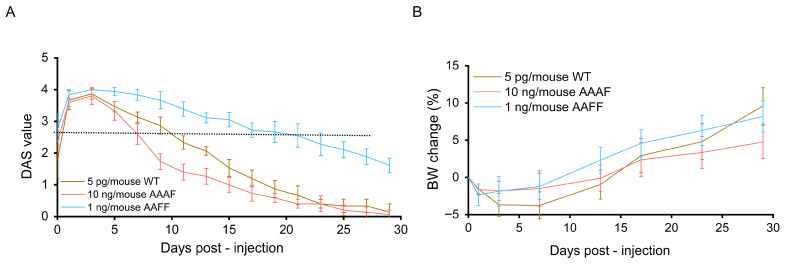
Evaluation of neuromuscular blockade and body weight changes in mice: (**A**) Neuromuscular blocking efficacy was assessed using the DAS assay over a 30-day period in mice treated with the rBoNT/A-WT and chimeric toxins (AAAF and AAFF). DAS scores were recorded at regular intervals to monitor the onset, progression, and recovery of muscle paralysis in each treatment group (n = 4–6). The dashed line indicates the approximate time of recovery onset from neuromuscular paralysis. (**B**) Body weight (BW) was monitored throughout the study and plotted as percentage change relative to baseline (Day 0). Data are presented as mean ± SD.

After intramuscular injection of each recombinant toxin into the murine gastrocnemius, neuromuscular paralysis was monitored for 30 days with the DAS assay. As shown in Figure 5A, all toxin-treated groups exhibited marked muscle paralysis. Recovery onset varied among groups: mice given rBoNT/A-WT by Day 10, whereas the AAFF group not until Day 21. The AAAF chimera induced near-complete paralysis yet triggered the earliest rebound, beginning on Day 7 and reaching substantial recovery by Day 29. Body weight trajectories showed only minor changes over the course of the study (Figure 5B), with all tested proteins causing approximately a 5% reduction relative to baseline and a maximum increase of approximately 10%. Notably, higher doses of rBoNT/A-WT (10 pg/mouse) are known to produce more pronounced effects on body weight [38]. These aspects, along with the current advancements in engineered chimeric botulinum neurotoxins, are summarized in Table 1.

In vivo assessment showed that AAAF and AAFF required approximately 100–1000-fold higher doses than rBoNT/A-WT to induce muscle paralysis, reflecting their extensive structural modifications and divergence from the native toxin architecture. Despite this reduced potency, both chimeras exhibited improved safety profiles: AAAF showed faster recovery from neuromuscular paralysis, whereas AAFF produced more prolonged paralysis, highlighting the functional impact of structural alterations on neuromuscular dynamics. However, the requirement for substantially higher doses may raise potential immunogenicity concerns, as BoNT/A-F chimeras would need larger doses to achieve therapeutic effects. A comparable situation has been reported for BoNT/B, which is administered clinically at a BoNT/A: BoNT/B ratio of approximately 1:50–100 and exhibits higher immunogenicity than BoNT/A [23]. Collectively, these findings suggest that receptor-binding domain recombination can modulate the duration of neuromuscular blockade, while also underscoring the need for further structural optimization—such as targeted domain refinement or linker adjustment—to balance efficacy and safety. Finally, although mice were used for practical and ethical considerations, DAS scoring is generally considered more precise in rats, and species-specific differences may contribute to variability or a slight underestimation of toxin activity [38].

### 2.5. Preliminary Analysis of Receptor-Binding Mechanism

To explore how structural alterations lead to functional changes, the receptor-binding domains of wild-type BoNT/A and the chimeric toxins were first constructed by homology modeling. The modeled receptor-binding domains were then docked separately with the two receptors of the established dual-receptor mechanism: ganglioside GT1b, mediating initial membrane tethering, and SV2A, responsible for high-affinity binding and toxin internalization [14]. Interface mapping identified residues potentially involved in receptor interactions, and binding free-energy calculations estimated the relative strengths of these interactions.

**Figure 6:**
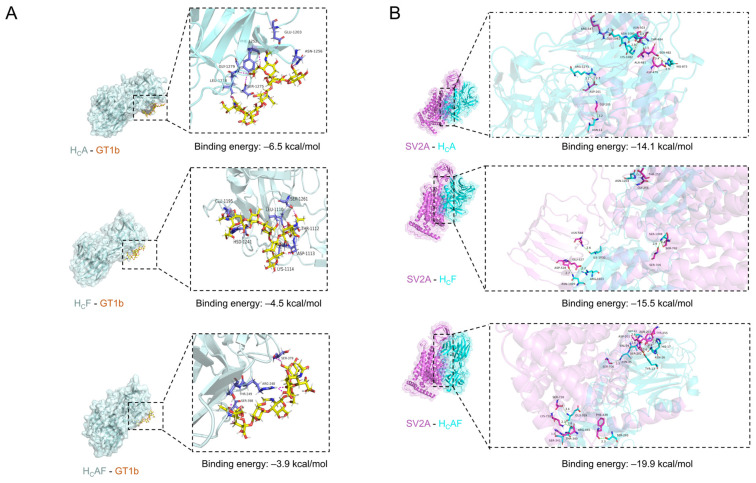
Mechanism analysis of the receptor binding: (**A**) Molecular docking models of H_C_A, H_C_F, and H_C_AF with the ganglioside GT1b. Left panels show overall 3D cartoon representations of the complexes with GT1b bound. Right panels highlight key residues forming hydrogen bonds with GT1b. Cyan indicates the receptor-binding domain, and yellow indicates GT1b. (**B**) Molecular docking models of H_C_A, H_C_F, and H_C_AF with the SV2A protein. Left panels present 3D cartoon structures of the receptor-binding domains interacting with SV2A. Right panels indicate key residues involved in forming hydrogen bonds between the toxin domains and SV2A. Pink indicates SV2A, while blue indicates the receptor-binding domain.

Polysialylated gangliosides and SV2A serve as dual receptors for BoNT/A uptake. Docking simulations compared the interactions of rBoNT/A-WT (H_C_A), AAAF (H_C_AF), and AAFF (H_C_F) with GT1b and SV2A (Figure 6). For GT1b, all domains used polar residues (Ser, Thr, and Asn) for hydrogen bonding with sialic acids, complemented by Glu, Trp, and Leu hydrophobic contacts. The H_C_A exhibited the most extensive hydrogen-bond network, including Asn1256, Ser1275, and Gly1279 (ΔG = −6.46 kcal/mol), whereas H_C_F showed moderate interactions via Lys1114 and Glu1195 (ΔG = −4.50 kcal/mol), and H_C_AF showed the weakest, with fewer key contacts (e.g., Arg1118, Ser1249; ΔG = −3.89 kcal/mol). For SV2A, conserved receptor residues Asp201, Tyr255, Thr257, and Ser728 formed hydrogen bonds with the toxins, while additional stabilization arose from toxin residues: H_C_A (His873, Gln1081, Lys1082, Asn1177, Arg1273; ΔG = −14.1 kcal/mol), H_C_F (Asn1020, Arg1022, Ile1030, Ser1088, Asn1253; ΔG = −15.5 kcal/mol), and H_C_AF (Tyr883, Asn886, His887, Gly901, Val904, Asn905, Ser1163, Glu1189, Thr1210; ΔG = −19.9 kcal/mol). The aforementioned complexes employ both hydrogen bonding and hydrophobic anchoring, with interaction strength variations correlating with predicted receptor affinity. While receptor-binding affinity appears to shape the pharmacodynamic profile, overall potency likely depends on the multifactorial in vivo environment. Additional structural factors, such as local backbone flexibility and receptor glycosylation, may further influence receptor recognition and warrant further experimental investigation.

## 3. Discussion

Domain recombination, particularly through receptor-binding domain engineering, provides a versatile approach to dissect how modular rearrangements influence the structure of BoNTs, receptor interactions, and cellular uptake. In this study, we designed, expressed, and characterized a panel of chimeric BoNTs, in which the receptor-binding segments from BoNT/B or BoNT/F were grafted onto the catalytic and translocation backbone of the BoNT/A. Two novel chimeric toxins—AAAF and AAFF—were designed and functionally characterized.

First, the AAAF and AAFF chimeric toxins were successfully expressed, although their yields were lower than that of the wild-type; nonetheless, the yields were still markedly higher than linker-free BoNT/A-B chimeras. Second, both AAAF and AAFF retained enzymatic activity in cell-free assays. In Neuro-2a cells, both AAAF and AAFF were efficiently internalized, with SNAP-25 cleavage efficiencies of ~46% for AAAF, ~44% for the rBoNT/A-WT, and notably higher at ~73% for AAFF, reflecting enhanced intracellular activity of the latter. Subsequently, in vivo assays showed that AAAF induced reversible muscle paralysis with a faster recovery than WT, whereas AAFF produced more sustained effects, and neither toxin caused substantial body weight changes at the tested doses. Overall, these data indicate that grafting of receptor-binding domains can modulate functional characteristics, yet the ultimate potency remains contingent upon both molecular compatibility and the multifactorial in vivo environment.

Incorporation of the BoNT/A-F combinations expands the diversity of engineered botulinum neurotoxins, enriching the current repertoire of chimeric toxins within domain engineering frameworks. Chimeric toxins (e.g., BoNT/B- or BoNT/E-derived variants) have been engineered to enhance neuronal targeting, optimize intracellular delivery, and refine efficacy and the duration of neuromuscular blockade by replacing the receptor-binding domain of BoNT/A with that of other serotypes [24,25,26,28,29,31]. However, BoNT/F—a donor candidate capable of binding all SV2 isoforms and sharing conserved ganglioside-binding motifs with BoNT/A—has been largely overlooked [30]. Moving beyond the conventional BoNT/A and BoNT/B or BoNT/E pairings, we introduce BoNT/F as the new donor module, enlarging the combinatorial space for toxin design. In this study, a series of novel BoNT/A-derived chimeric toxins was constructed by replacing the receptor-binding domains portions (H_CN_, H_CC_, or H_C_) with the cognate segment from BoNT/B or BoNT/F. Among the six constructs produced, AAAF and AAFF emerge as soluble and correctly processed holotoxins, demonstrating the structural compatibility between BoNT/A and BoNT/F domains and validating that the overall architecture remains intact after domain exchange. Unlike the BoNT/A-B chimeras, AAAF and AAFF expressed as soluble proteins in *E. coli* without extra-flexible linkers, whereas AAAB and AABB showed very low expression, likely due to codon bias and misfolding in the bacteria. Together, these results demonstrate the modular flexibility of BoNTs and validate their potential as a platform for engineering neurotoxins with modified therapeutic profiles.

The BoNT/A-F chimeras exhibited measurable differences in cellular uptake, pharmacodynamics, and toxicity, suggesting that domain substitutions can influence functional properties. Previous studies showed that BoNT/A binds synaptosomes far more weakly than other serotypes—only 12% of the affinity compared to 34–42% for BoNT/B, BoNT/E, and BoNT/F [26]. Another research developed AB and AE chimaeras by grafting the H_C_ domain of BoNT/B or BoNT/E onto BoNT/A, yielding constructs with an optimized SNAP-25 cleavage and in vivo neuromuscular effects [24,28,31]. Nevertheless, whether BoNT/A-F chimeras can achieve a favorable balance of potency, duration, and safety remains unclear. Here, we built two BoNT/A-F chimeras—AAAF and AAFF—and compared them with rBoNT/A-WT. In cell-free assays, both chimeras retained enzymatic activity, with AAFF comparable to the WT and AAAF slightly reduced. In Neuro-2a cells, both chimeras efficiently entered neurons and cleaved SNAP-25 (~46% for AAAF, ~44% for WT, ~73% for AAFF), indicating enhanced intracellular activity for AAFF, although the absence of neuron-specific components and a complete ganglioside repertoire likely underestimated uptake. Paradoxically, both chimeras required substantially higher in vivo doses to induce neuromuscular block, confirming their reduced potency—a trend observed in earlier chimeras and likely reflecting altered receptor interactions or pharmacokinetic profiles [24,28]. Despite reduced potency, AAFF induced the longest-lasting paralysis, whereas AAAF showed faster recovery, and neither caused substantial body weight changes, reflecting favorable systemic safety. Nonetheless, the need for higher doses may increase systemic exposure and potential immunogenicity. Overall, AAAF and AAFF exhibit tunable pharmacodynamic profiles with limited systemic effects, but their reduced in vivo potency and potential immunogenicity remain important considerations for translational development.

Collectively, these findings establish BoNT/A-F chimeric toxins as versatile platforms, validating receptor-binding domain grafting as a robust strategy for retargeting toxin behavior. However, functional characterization in Neuro-2a cells—which express SV2A yet lack complex gangliosides—suggests SV2-mediated internalization predominates in this simplified membrane environment [34,35]. Consequently, the altered receptor repertoire may compromise stringent selectivity assessments, constraining the predictive validity of in vitro affinity data for physiological contexts. Moreover, molecular modeling together with the relatively low-affinity interactions observed in Neuro-2a cells may inadequately recapitulate the complex, high-affinity, and high-potency behavior of these toxins at motor nerve terminals in vivo. Nonetheless, BoNT/A-F chimeras still retain significant translational potential. Notably, for wild-type BoNT/A, a relatively small increase in dose (e.g., from 5 pg/mouse to 10 pg/mouse) is sufficient to induce a pronounced and readily measurable reduction in body weight, indicating that biological effects can change markedly within a narrow dosing range. In contrast, the attenuated potency of these chimeric constructs may expand the therapeutic window, thereby enhancing dosing flexibility and safety margins. In addition, the preserved bioactivity, together with divergent recovery profiles, suggests that receptor-domain substitution may modulate both potency and pharmacodynamic behavior, enabling more tailored functional outcomes. Overall, these findings support further exploration of BoNT/A-F chimeric toxins.

## 4. Conclusions

This study designed and expressed novel BoNT/A-F chimeras and performed a preliminary evaluation of their in vitro and in vivo functional activities. These results suggest that receptor-binding domain engineering may influence pharmacological properties and may serve as a basis for next-generation neuromodulator development. Although these findings provide preliminary insights, several limitations remain. For example, the Neuro-2a cells, though convenient, lack neuronal subtype fidelity, and our molecular docking relied on static snapshots that ignore conformational flexibility and physiological post-translational modifications. Future work will integrate primary neurons and target receptor mutagenesis to deepen mechanistic insight and guide the development of safer, more potent BoNT therapeutics.

## 5. Materials and Methods

### 5.1. Strains, Plasmids, and Chemicals

The strains and plasmids used in this study are listed in Table S2. *Escherichia coli* DH5α (for cloning) and *E. coli* BL21 (DE3) (for expression) were purchased from TsingKe Biotech (Beijing, China). Codon-optimized genes encoding BoNT/A, BoNT/F H_CN_F and H_CC_F, and BoNT/B H_CN_B and H_CC_B were synthesized by Genewiz (Nanjing, China). The primers were synthesized by Sangon Biotech (Shanghai, China). Enzymes, cloning reagents, and the following reagents were obtained from ThermoFisher (Waltham, MA, USA): Pierce BCA Protein Assay Kit, Trypsin, ProLong^TM^ Diamond Antifade Mountant with 4′, 6-diamidino-2-phenylindole (DAPI), and SuperSignal^TM^ West Pico PLUS substrate. Antibodies were acquired from the following suppliers: mouse anti-His antibody (Proteintech, Wuhan, China, 66005-1-Ig); rabbit anti-SV2A antibody (Abcam, Cambridge, UK, ab32942), rabbit anti-SNAP-25 antibody (Abcam, EPR3275); HRP-conjugated goat anti-mouse IgG(H + L), HRP-conjugated goat anti-rabbit IgG(H + L), HRP-conjugated anti-GAPDH antibody, CoraLite488-conjugated goat anti-mouse IgG(H + L), and CoraLite594-conjugated goat anti-rabbit IgG(H + L) (Proteintech, China). Chemicals were obtained from Macklin (Shanghai, China), and the recombinant trypsin inhibitor from soybean was purchased from Sangon Biotech (Shanghai, China).

### 5.2. Molecular Cloning

All plasmids were constructed by the Gibson assembly method using the primers listed in Table S3 [40]. The recombinant wild-type BoNT/A (rBoNT/A-WT) and each chimeric variant were constructed with a C-terminal 6 × His-tag and cloned into vector pET-28a. Domain swaps were introduced as follows: AAAB and AAAF were generated by substituting the BoNT/A H_CC_ domain with that of BoNT/B or BoNT/F, respectively; AABA and AAFA were generated by replacing the BoNT/A H_CN_ domain with the BoNT/B or BoNT/F H_CN_; AABB and AAFF were generated by full replacement of the entire H_C_ domain with the BoNT/B or BoNT/F H_C_. In addition, a flexible GGGGS linker composed of Gly-Gly-Gly-Gly-Ser was incorporated at domain junctions to facilitate proper folding and expression when necessary.

### 5.3. Protein Expression and Purification

Seed cultures of the engineered strains were transferred into Terrific Broth (TB) medium supplemented with 50 µg/mL kanamycin at 37 °C. Once an OD_600_ reached 0.6–0.8, a final concentration of 0.5 mM Isopropyl β-D-thiogalactoside (IPTG) was added for inducing protein expression, and the culture was sustained for 24 h at 16 °C. The harvested cells were resuspended in Buffer A (20 mM Tris-HCl, 500 mM NaCl, pH 8.5) and lysed via high-pressure homogenization and centrifuged at 12,000 rpm for 30 min for filtration. The supernatant was purified using a Ni-NTA column, with target proteins eluted by stepwise imidazole gradient in Wash Buffer B (20 mM Tris-HCl, 500 mM NaCl, 500 mM imidazole, pH 8.5). Eluted fractions were dialyzed against SEC running buffer (20 mM Tris-HCl, 150 mM NaCl, 2.5 mM CaCl_2_, pH 7.6), aliquoted, and stored at −20 °C [41]. Protein concentration was quantified using the Pierce BCA Protein Assay Kit and converted to molarity based on molecular weight.

### 5.4. Proteolytic Activation of Recombinant Toxins

Single-chain precursors were nicked to the active di-chain form by limited trypsinolysis. Purified toxin (the rBoNT/A-WT or chimera) was incubated with trypsin at rBoNT/A: trypsin mass ratios ranging from 250:1 to 50:1 (*w*/*w*) in the SEC running buffer (37 °C, 60 min). After digestion, trypsin was inactivated by the addition of trypsin inhibitor (trypsin: trypsin inhibitor = 1:10, *w*/*w*), and the samples were analyzed by sodium dodecyl sulfate polyacrylamide gel electrophoresis (SDS-PAGE) analysis. For the SDS-PAGE analysis, non-reducing samples (Ox.) were loaded directly in 5 × SDS loading buffer to maintain disulfide bonds, whereas the reducing samples (Red.) were supplemented with 5 × SDS loading buffer containing 5% β-mercaptoethanol and heated at 95 °C for 10 min to fully disrupt disulfide linkages. Activation efficiency was assessed by monitoring the conversion of the single-chain precursor (150 kDa) into the activated di-chain form (LC: 50 kDa; HC: 100 kDa), while minimizing non-specific degradation. The optimized proteolytic protocol was then applied uniformly to all chimeric toxins prior to functional assays. To further verify the activation products, Western blot analysis was performed using mouse anti-His antibody (1:10,000) as the primary antibody and HRP-conjugated goat anti-mouse IgG(H + L) (1:10,000) as the secondary antibody.

### 5.5. Cell-Free rSNAP-25 Proteolysis Assay

The substrate peptide rSNAP-25, expressed in *E. coli* with a C-terminal 6 × His-tag, was purified to homogeneity using nickel affinity chromatography. The rBoNT/A-WT and chimeric toxins were prepared at 0.01 mg/mL and then serially diluted in assay buffer (50 mM Tris-HCl, 20 μM ZnCl_2_, 10 mM DL-Dithiothreitol, pH 7.6). To assess concentration-dependent cleavage, reactions of equal volume containing varying concentrations (0.05–40 nM) of WT, AAAF, or AAFF and 2.4 μM rSNAP-25 in assay buffer were incubated at 37 °C for 30 min. Reactions were terminated by adding 10 μL of 5 × SDS-PAGE loading buffer to 40 μL of the mixture, followed by heating at 95 °C for 5 min. Products were resolved on 12% SDS-PAGE, and cleavage efficiency was quantified using ImageJ software (version 1.53k; National Institutes of Health, Bethesda, MD, USA) by normalizing the cleaved SNAP-25 signal to the total SNAP-25 signal (full-length plus cleaved) in the same lane.

### 5.6. Cellular Internalization of Chimeric Toxins

To assess the cellular uptake of chimeric toxins, the mouse Neuro-2a cells (Baidi Biotech, Hangzhou, China) were subjected to immunofluorescence analysis. Cells were cultured in Dulbecco’s Modified Eagle Medium (DMEM; Gibco, Grand Island, NY, USA) supplemented with 10% fetal bovine serum and 100 U/mL penicillin–streptomycin at 37 °C in a humidified incubator with 5% CO_2_. Immunofluorescence staining was performed as described previously, with minor modifications [42]. When 80% confluency was reached (~48 h), cells on 14 mm glass coverslips in 24-well plates were exposed to proteolytically activated wild-type and chimeric toxins (WT, AAAF, AAFF; 10 nM) in 500 µL of serum-free DMEM for 12 h at 37 °C. After incubation, cells were washed three times with phosphate-buffered saline (PBS) and fixed with ice-cold methanol (−20 °C) for 5 min.

Fixed cells were blocked with 5% goat serum for 1 h at room temperature, then incubated overnight at 4 °C with mouse anti-His antibody (1:500) and rabbit anti-SV2A antibody (1:200) in PBST containing 1% bovine serum albumin (BSA). After washing three times with PBS, cells were labeled for 1 h in the dark at room temperature with CoraLite 488-conjugated anti-mouse (1:300) and CoraLite 594-conjugated anti-rabbit (1:300) secondary antibodies (1% BSA in PBST). Coverslips were mounted in ProLong^TM^ Diamond Antifade Mountant with DAPI for nuclear counterstaining. Images were captured on a N-STORM super-resolution microscope (Nikon, Tokyo, Japan) with excitation wavelengths of 488 nm (His-tag), 594 nm (SV2A), and the DAPI channels. Additionally, cells treated under the same conditions were lysed, and the extracted proteins were used for subsequent analyses with anti-His antibody.

### 5.7. Enzymatic Activity in Neuro-2a Cells

Western blot analysis was performed to semi-quantitatively evaluate toxin-mediated cleavage of endogenous SNAP-25 in Neuro-2a cells. Cells were seeded in 12-well plates, grown to ~80% confluency for 24 h, and treated with 10 nM of the activated di-chain wild-type or chimeric toxins for 12 h to assess SNAP-25 cleavage. After treatment, cells were lysed in the Radio Immunoprecipitation Assay lysis buffer (RIPA) with protease inhibitors, and lysates were clarified by centrifugation (10,000× *g*, 10 min, 4 °C).

Proteins were resolved by 12% SDS-PAGE, transferred to nitrocellulose, and blocked with 5% non-fat milk in TBST (1 h, RT). Membranes were probed overnight at 4 °C with rabbit monoclonal anti-SNAP-25 antibody (1:5000), followed by HRP-conjugated goat anti-rabbit IgG(H + L) (1:10,000; 45 min, RT). Signals were developed with SuperSignal™ West Pico PLUS substrate and imaged with a ChemiScope 3300 system (Qinxian, Shanghai, China). After stripping, membranes were re-probed with HRP-conjugated anti-GAPDH antibody (1:10,000) for normalization. The SNAP-25 cleavage was quantified by ImageJ-based densitometry; band intensities were normalized to GAPDH and expressed as percent cleavage relative to untreated controls.

### 5.8. Mouse Digit Abduction Score (DAS) Assay

All animal protocols were reviewed and approved by the Institutional Animal Care and Use Committee of South China University of Technology (Approval No. 2025017). Male C57BL/6 mice (8–12 weeks, 20–27 g) were screened for normal baseline responses and randomly assigned to weight-balanced groups. Under 3% isoflurane in oxygen, 20 μL of test toxins (diluted in sterile saline + 0.5% BSA) was delivered into the right gastrocnemius-soleus muscle via a 30-gauge needle. Paralytic potency was evaluated by Mouse Digit Abduction Score (DAS) assay as previously described [36].

All proteins used were in the trypsin-activated di-chain form. Doses were chosen from the literature for the rBoNT/A-WT (5 pg). For AAAF and AAFF, an ascending series from 20 pg to 20 ng/mouse was screened to identify the lowest paralytic dose. Once established, neuromuscular potency and safety were confirmed in cohorts of 4–6 mice. Paralysis was quantified daily with a validated 5-point DAS scale (0 = normal; 4 = complete flaccidity); each mouse was scored in triplicate at every observation for consistency. Body weight (BW) and general health were recorded continuously.

### 5.9. Molecular Docking with GT1b and SV2A

The 3D structure of GT1b was extracted from the BoNT/A receptor-binding domain (H_C_A) co-crystal complex (PDB: 2VU9), and the BoNT/F receptor-binding domain (H_C_F) from PDB: 3FUQ. The receptor-binding domain of the AAAF chimera (H_C_AF) was modeled with Swiss-Model (https://swissmodel.expasy.org/) [43]. GT1b was docked to H_C_A, H_C_F, and H_C_AF in LeDock v1.0 (http://www.lephar.com) using rigid-body scoring. SV2A (PDB: 8JLC) was subsequently docked to each domain with GRAMM (http://vakser.compbio.ku.edu/resources/gramm/), also in rigid mode [44]. All structures were visualized using PyMOL (v2.5, Schrödinger, LLC, New York, NY, USA).

### 5.10. Statistical Analysis

All experimental data were analyzed using one-way ANOVAs in OriginPro 2023. One-way ANOVA was applied to compare biological activities across different protein variants. For comparisons between tagged and untagged protein groups, *t*-tests were used. The results are presented as mean ± standard deviation, and a *p*-value < 0.05 was considered statistically significant.

## Figures and Tables

**Table 1:** Research progress on chimeric modified botulinum toxin.

Chimera Notation	Domain Composition	Performance Improvement	Reference
chimera AE	/A LC-H_N_, /E H_C_	Persistent muscle weakness (up to 37 days)	[24]
chimera EA	/E LC-H_N_, /A H_C_	Therapeutic efficacy against chronic pain	[25]
LC/E-BoNT/A	/E LC, /A full length	Long-acting anti-hyperalgesic	[31]
AABB AAAB	/A LC-H_N_, /B H_C_ /A LC-H_N_-H_CN_, /B H_CC_	4-fold higher potency than BoNT/A in vitro	[26]
chimera AB chimera BA	/A LC-H_N_, /B H_C_ /B LC-H_N_, /A H_C_	Chimaera AB: prolonged paralysis (up to 50 days); Chimaera BA: block exocytosis in both neurons and SNAP-25-deficient cells	[28]
AABB	/A LC-H_N_, /B H_C_	Higher potency in vitro; similar to wild-type in vivo	[29]
AAFF AAAF	/A LC-H_N_, /F H_C_ /A LC-H_N_-H_CN_, /F H_CC_	Both show improved safety; AAAF recovers faster, AAFF lasts longer	This study

## Data Availability

The data that support the findings of this study are available on request from the corresponding author.

## Supplementary Materials

The following supporting information can be downloaded at: https://www.mdpi.com/article/10.3390/toxins18050205/s1, Figure S1: Optimization of induction conditions and expression level analysis of the rBoNT/A-WT; Figure S2: Comparison of expression and purification profiles between the rBoNT/A-WT and chimeric toxins; Figure S3: SDS-PAGE analysis on the purified rSNAP-25; Table S1: Expression and purification summary of rBoNT/A-WT and BoNT/A-F chimeras; Table S2: Strains and plasmids used in this study; Table S3: All primers used in this study; Table S4: Gastrocnemius injection dosage screening.
