# Supplementary material for: Design and Characterization of Recombinant and Chimeric BoNT/A Neurotoxins with Receptor-Binding Domain Grafting
Source: Toxins (Basel). 2026 Apr 29;18(5):205. doi: 10.3390/toxins18050205 (PMC13211482; doi:10.3390/toxins18050205)
Supplement: Supplementary file 1 [file toxins-18-00205-s001.zip › toxins-4264705-supplementary.pdf]

Supplementary Materials: Design and Characterization of Recombinant and Chimeric BoNT/A Neurotoxins with Receptor Binding Domain Grafting

Sihan Pan [1], Yuanzhi Ye [1], Yang Li [1], Hongxin Fu [1] and Jufang Wang [1,2,*]

This supplementary material contains 3 figures and 4 tables.

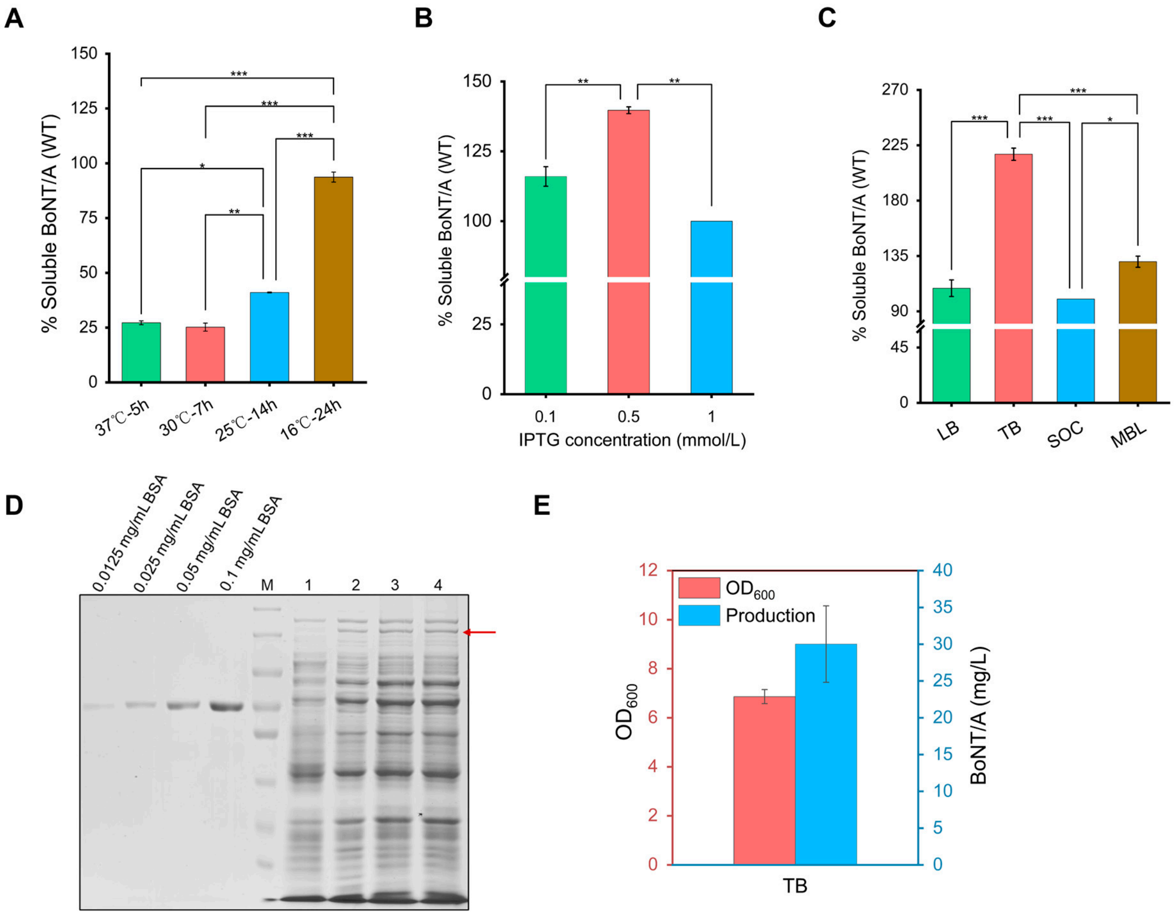

Figure S1. Optimization of induction conditions and expression level analysis of the rBoNT/A-WT. (A) Induction temperature. The cultures were incubated at 16 °C for 24 h, 25 °C for 14 h, 30 °C for 7 h, and 37 °C for 5 h. Protein expression levels were quantified by the SDS-PAGE, with the 16 °C condition set as 100%; (B) IPTG concentration. A final concentration of 0.2, 0.5, or 1 mM IPTG was added. Expression at 1 mM was set as 100%; (C) The medium. Four different media (LB, TB, MBL, SOC) was selected for evaluating, with LB medium as the reference (100%). (D) SDS-PAGE analysis under the optimal induction condition (0.5 mM IPTG, 16 °C, TB). Lane M: marker; Lane 1: uninduced control; Lanes 2-4: three biological replicates of induced lysate supernatants. BSA standards were run in parallel and the arrow indicates the target protein. (E) Quantification of protein expression based on densitometric analysis of SDS-PAGE bands under optimal conditions. Each experiment was performed in three independent repeats. Data are presented as mean ± standard deviation (SD), with statistical analysis by one-way ANOVA. Statistical significance is indicated as: $0.01 < {}^{*}p \leq 0.05$, $0.001 < {}^{**}p \leq 0.01$, ${}^{***}p < 0.001$.

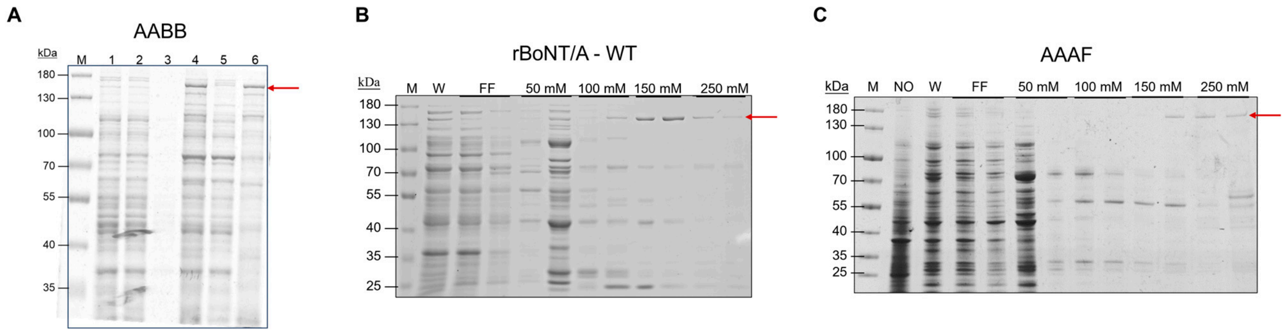

Figure S2. Comparison of expression and purification profiles between the rBoNT/A-WT and chimeric toxins. (A) SDS-PAGE analysis of AAAB expression induced under optimized conditions. M: marker; lanes 1-3: total, soluble, and insoluble fractions without IPTG induction; lanes 4-6: total, soluble, and insoluble fractions following IPTG induction. (B-C) Purification profiles of the rBoNT/A-WT and AAAF. M: marker; Lane NO: uninduced sample; Lane W: whole-cell lysate prior to purification; Lane FF: flow-through fraction; 50-500 mM: imidazole-eluted fractions.

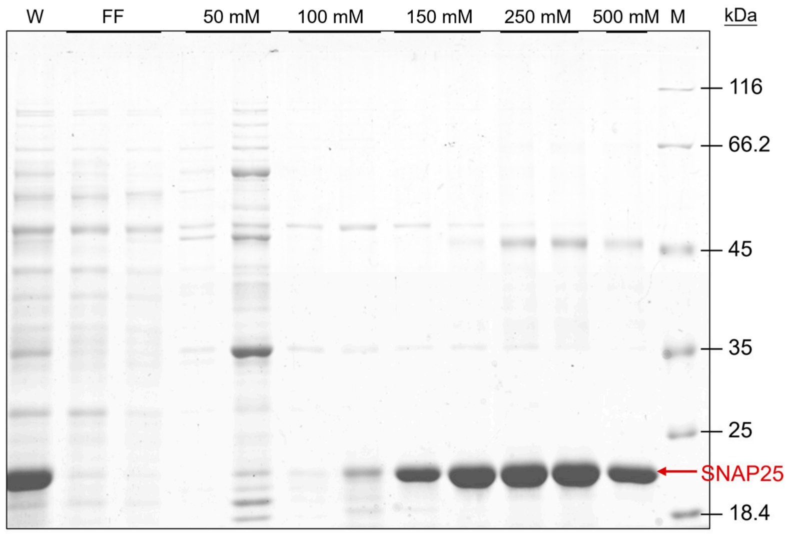

Figure S3. SDS-PAGE analysis on the purified rSNAP-25. SDS-PAGE analysis showing whole-cell lysate before purification (Lane W), flow-through fraction (Lane FF), and imidazole-eluted fractions (50-500 mM). M: molecular weight marker; red arrow indicates the target rSNAP-25 band (~25 kDa).

Table S1. Expression and purification summary of rBoNT/A-WT and BoNT/A-F chimeras

Construct	Culture Volume (L)	Titer [a] (mg/L)	Concentration [b] (mg/mL)	Purity [c] (%)	Estimated Yield (mg/L, 15 mL eluate)
rBoNT/A-WT	0.2	30.0 ± 5.20	0.13 ± 0.035	92.7 ± 2.44	9.04
AAAF	0.5	14.8 ± 0.61	0.04 ± 0.012	89.9 ± 2.21	1.08
AAFF	0.5	14.7 ± 0.92	0.05 ± 0.013	88.6 ± 1.90	1.33

[a, c] Titer and purity were semi-quantified by densitometric analysis of SDS-PAGE bands using ImageJ.

[b] Protein concentration was measured using the BCA protein assay.

Table S2. Strains and plasmids used in this study

Strains/plasmids	Genotype or relevant	Source/Ref
**Strain**		
E. coli DH5α	Plasmid cloning	Lab Stock
E. coli BL21 (DE3)	Protein expression	Lab Stock
**Plasmid**		
pET28a	T7 promotor	Lab Stock
pET28a-BoNT/A	BoNT/A	This study
pET28a-SNAP-25	SNAP-25	This study
pET28a-H_{CN}F	H_{CN}F	This study
pET28a-H_{CC}F	H_{CC}F	This study
pET28a-H_{CN}B	H_{CN}B	This study
pET28a-H_{CC}B	H_{CC}B	This study

Table S3. All primers used in this study

**Primer**	**Sequences（5'-3'）**
BoNT/A-F	CCGCGCGGCAGCCATATGATGCCTTTTGTAAATAAGCAATTTAA
BoNT/A-R	TGGTGGTGGTGGTGCTCGAGTAAAGGACGTTCTCCCCATC
BoNT/A-VF	CTCGAGCACCACCACCACCAC
BoNT/A-VR	CATATGGCTGCCGCGCGGCAC
T7	AGATATACCATGGGCAGCAG
T7 TER	CCTTTCGGGCTTTGTTAGCAG
AAAB-NF	TTATACGACAATCAATCTTATTCAGAATACCTTAAAGACTTCTG
AAAB-NR	GTGGTGGTGGTGCTCGAGTTCCGTCCATCCTTCGTC
AAAF-NF	TTATACGACAATCAATCTGATCCATCAATACTTAAAGACTTCTG
AAAF-NR	GTGGTGGTGGTGCTCGAGGTTTTCCTGCCAACCGTG
AABA-NF	TTATTATCAACGTTCACGGAGTACATCAGTGAAATCCTAAATAATATAATATTAAAC
AABA-NR	TCTTTTAAAATTCCAGAATTCGACTGTATTTTATAACGTTCTTC
AAFA-NF	TTATTATCAACGTTCACGGAGTACATCAAAAAGATCAAAGATAATTCAATACTAG
AAFA-NR	AGAACCACCACCACCCGGTTCGTCTGAGTAGAGGG
AAAX-CF	CTCGAGCACCACCACCAC
AAAX-CR	AGATTGATTGTCGTATAAGTCTTTAATC
AABA-CF	AATTCTGGAATTTTAAAAGATTTTT
AAFA-CF	AACCGGGTGGTGGTGGTTCTAATTCTGGAATTTTAAAAGATTTTT
H_{CN}F-R	CGGTTCGTCTGAGTAGAGGG
H_{CC}F-F	CCCTCTACTCAGACGAACCGGATCCATCAATACTTAAAGACTTCTG
Overlap-H_{CN}B-F	ACATCGGTGGTGGTGGTTCTAGTGAAATCCTAAATAATATAATATTAAAC
H_{CN}B-R	CGACTGTATTTTATAACGTTCTTCG
H_{CC}B-F	AACGTTATAAAATACAGTCGTATTCAGAATACCTTAAAGACTTCTG
AA-CF	CTCGAGCACCACCACCACCAC
AA-CR	TCCGTGAACGTTGATAATAAACGCTGATTGTCTACATATTTTGA

Table S4 Gastrocnemius injection dosage screening

Drug delivery protein	Dosage	Average DAS after 8 h
AAAF	20 pg/mouse	0
	200 pg/mouse	0
	1 ng/mouse	2.3
	10 ng/mouse	3.7
	20 ng/mouse	4.0
AAFF	20 pg/mouse	0.3
	200 pg/mouse	1.3
	500 pg/mouse	2.3
	1 ng/mouse	3.7
	2 ng/mouse	4.0
